# Increased *TPSAB1* Copy Number in a Family With Elevated Basal Serum Levels of Tryptase

**DOI:** 10.3389/fmed.2021.577081

**Published:** 2021-04-13

**Authors:** Laura Hernández-Hernández, Catalina Sanz, Elena Marcos-Vadillo, Asunción García-Sánchez, Esther Moreno, Félix Lorente, David González-de-Olano, Ignacio Dávila, María Isidoro-García

**Affiliations:** ^1^Department of Allergy, University Hospital of Salamanca, Salamanca, Spain; ^2^Institute for Biomedical Research of Salamanca (IBSAL), Salamanca, Spain; ^3^Department of Microbiology and Genetics, University of Salamanca, Salamanca, Spain; ^4^Department of Clinical Biochemistry, University Hospital of Salamanca, Salamanca, Spain; ^5^Department of Biomedical and Diagnostics Sciences, University of Salamanca, Salamanca, Spain; ^6^Department of Allergy, Instituto Ramón y Cajal de Investigación Sanitaria (IRYCIS), University Hospital Ramón y Cajal, Madrid, Spain; ^7^Department of Medicine, University of Salamanca, Salamanca, Spain

**Keywords:** tryptase, β-tryptase, hereditary α-tryptasemia, mast cells, basal serum tryptase

## Abstract

**Background:** Some recent familial studies have described a pattern of autosomal dominant inheritance for increased basal serum tryptase (BST), but no correlation with mRNA expression and gene dose have been reported.

**Objective:** We analyzed *TPSAB1* mRNA expression and gene dose in a four-member family with high BST and in two control subjects.

**Methods:** Blood samples were collected from the family and control subjects. Complete morphologic, immunophenotypical, and molecular bone marrow mast cell (MC) studies were performed. mRNA gene expression and gene dose were performed in a *LightCycler 480 instrument*. Genotype and CNV were performed by quantitative real-time digital PCR (qdPCR).

**Results:** CNV analysis revealed a hereditary copy number gain genotype (3β2α) present in all the family members studied. The elevated total BST in the family members correlated with a significant increase in tryptase gene expression and dose.

**Conclusions and Clinical Relevance:** We present a family with hereditary α-tryptasemia and elevated BST which correlated with a high expression of tryptase genes and an increased gene dose. The family members presented with atypical MC-mediator release symptoms or were even asymptomatic. Clinicians should be aware that elevated BST does not always mean an MC disorder.

## Introduction

Mast cells (MCs) are tissue effector cells that participate in several physiologic and pathologic processes such as innate immunity, immunomodulation, allergy, autoimmunity, and neuroinflammation (1). Upon activation, MCs release preformed molecules stored in secretory granules, newly synthetized mediators, and several cytokines. On the stored proteases tryptase is a serine protease related to trypsin. Baseline serum tryptase (BST) levels correlate with MC burden and/or MC number in acute allergic reactions, thus providing a measure of MC activation (2).

Mast cell disorders (MCD) can be associated to an increased number of mast cells, activation of MCs, or both. A recent consensus (3) has classified MC activation (MCA) disorders in primary mast cell activation syndrome (MCAS)—when clonality is present—secondary MCAS, and idiopathic MCAS. The criteria indicative of systemic MCA are: (a) typical clinical signs and symptoms, (b) substantial and transient increase in an MC-derived mediator in biological fluids (being BST during or shortly after the acute event) compared to a baseline recorded either before the acute event or at least 24 h after all clinical signs and symptoms of the event have completely resolved, and (c) an objective major response of clinical symptoms to agents that attenuate the production or activity of MC-derived mediators (3). Recently, hereditary α-tryptasemia (HαT), which is associated with increased copy numbers of the *TPSAB1* gene encoding α-tryptase, has been included in the last consensus report of MCD (4).

The tryptase comprises different proteases, so far α, β, γ, and δ, which show minor differences in terms of their enzymatic properties (5). To date, the best characterized tryptase is the **β-tryptase**. Three β-tryptase subtypes have been described (βI, βII, and βIII). β-tryptase is stored in its active form, though it is maintained with little or no activity within the secretory granules due to the low pH value and stabilization mediated by other proteins. In the absence of the factors needed for stabilization, the structure dissociates into monomers that were initially considered to be inactive, though several studies have demonstrated the existence of active β-tryptase monomers (6). Compared to β-tryptase, **α-tryptase** is in low levels in the circulation, without degranulation. Both enzymes have a 93–96% similarity in their amino acid sequences. In addition, other tryptases have been described: **γ-tryptase**, which is less related to other tryptases and **δ-tryptase**, which is similar to α-tryptase in its amino acid sequence and is also constitutively secreted.

A region containing the human tryptase locus has been described in chromosome 16p13.3, containing at least four genes (7). The *TPSG1* gene, encoding for γ-tryptase, is followed by the *TPSB2* gene, which encodes for tryptases βII/βIII, and by the *TPSAB1* gene, which encodes for α-tryptase and β-tryptase. Lastly, the *TPSD1* gene encodes for δ-tryptase. Although the genes encoding for tryptases βI and βIII have been previously localized within the aforementioned chromosome, there is some controversy regarding their precise position. CpG islands have been identified in this region, a fact that complicates the cloning and sequencing processes (7).

Familial occurrence of increased serum BST has been rarely described. Lyons et al. (8) reported that increases in the *TPSAB1* gene, which codes for α-tryptase, were associated with BST levels >8 ng/mL. This trait is inherited, and the condition is called hereditary α-tryptasemia (HαT). We had the opportunity to study four members of a family with elevated BST for 16 years (2003–2019) which correlated with a high expression of tryptase and gene dose.

## Patients and Methods

### Ethics

The study was approved by the local Ethics Committee of the University Hospital of Salamanca (PI120913) and an informed consent form was signed in all cases.

### Subjects and Clinical Assessments

The index case was a patient in her fifties who in 3 years presented six events, without identifying known triggers, consisting of sudden episodes of shivering, abdominal pain, nausea, vomiting, and diarrhea, followed by muscular cramps; no cutaneous lesions, hypotension, or other symptoms appeared. The patient required hospitalization for these episodes, but symptoms resolved with supportive therapy. The index case had been studied by Internal Medicine where gastrointestinal, thyroid, suprarenal or celiac disease, carcinoid syndrome, neuroendocrine syndromes, autoimmunity, and porphyria were ruled out. The patient was then referred to the Department of Allergy. Physical exploration was unremarkable. Skin prick tests to aeroallergens and food extracts were negative. Total serum IgE was 21.6 kU/L, C3, C4, CH100, and C1-INH levels were normal, and parasite infections were ruled out. BST was 47.4 ng/ml (Thermo Scientific Phadia AB, Uppsala, Sweden). When repeated 2 months later, BST showed a value of 48.4 ng/ml. A bone marrow (BM) study was later performed strictly following the proposed criteria (9): BM MCs morphology was analyzed in toluidine blue and May-Grünwald-Giemsa-stained smears (10) and disclosed no abnormalities. Immunophenotypical analysis of CD25 expression on BM MCs was performed by flow cytometry using a multiparameter 4-color immunofluorescence technique according to consensus procedures and criteria previously defined by the Spanish Network on mastocytosis (REMA) (11, 12). Flow cytometry showed 0.006% of MCs on BM and <1/10^5^ cells on peripheral blood. Mast cells showed a normal phenotype (CD117++, CD45+, CD25–, CD2–, CD69^low^, and IgE+). Study of somatic activating codon Asp816-Val *KIT* mutation or other *KIT* mutations was performed in genomic DNA from fluorescence-activated cell-sorting purified populations of BM mast cells, neutrophils, eosinophils, monocytes, lymphocytes, CD34^+^ hematopoietic progenitor and precursor cells, and nucleated red cells, as described elsewhere (13). No mutations were detected. Meanwhile, antihistamines were prescribed on a daily basis, but six new episodes appeared. During one of these episodes, acute serum tryptase could be determined, showing no significant increase (35.9 ng/ml), even when considering the rule of an increase in BST exceeding 20% of baseline value plus absolute 2 ng/ml. The patient could fulfill one of the diagnostic criteria of MCAS (3), although clinical symptoms were not typical. Nevertheless, she had permanently increased BST without variation during acute episodes—although the participation of other putative mediators cannot be excluded—and she did not respond to anti-mast cell mediator treatment and, thus, a MCAS diagnosis was discarded. After these initial episodes, the index case has been asymptomatic up to date. In January 2019, her tryptase levels were 38.8 ng/ml.

**Figure 1:**
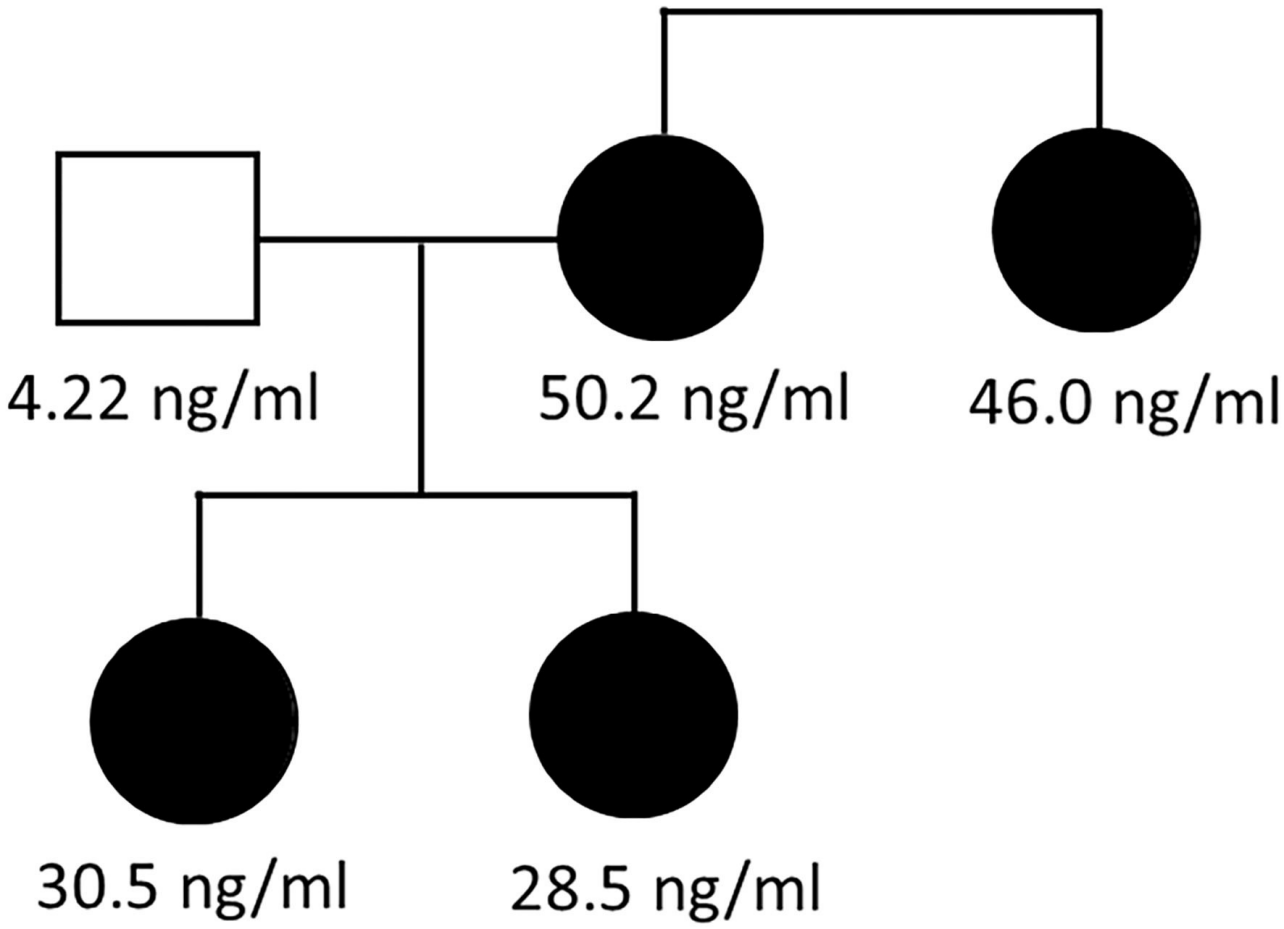
Family pedigree. Family pedigree showing hereditary alpha-tryptasemia syndrome. Numbers indicate serum baseline tryptase concentration (in ng/ml) in the index case (I.2), her sister (I.3), and two daughters (II.1 and II.2); values were normal in her husband (I.1).

We carried out a determination of BST levels and genetic analysis to the index case, her sister, her two daughters, and her husband. All of them gave informed written consent to perform these procedures. Serum baseline tryptase levels were as follows: 50.2 ng/ml (index case, I.2); 30.5 ng/ml (daughter #1, II.1); 28.5 ng/ml (daughter #2, II.2), 46 ng/ml (sister, I.3), and 4.22 ng/ml (husband, I.1; Figure 1). The sister and the two daughters were completely asymptomatic, as well as the husband, so no BM biopsy was performed. Furthermore, the index case and his family has been followed for 14 years and they have all remained asymptomatic up to date. Two individuals were included as controls for the expression and gene dose analyses. The inclusion criteria were (i) no symptoms or history of allergy; (ii) no symptoms or history of asthma or other pulmonary diseases; (iii) negative skin prick tests to a battery of common aeroallergens; (iv) absence of first-degree relatives with a history of asthma or atopy; and (v) BST lower than 11.4 ng/ml.

### Gene Expression Analysis

RNA was obtained with the RiboPure^TM^-Blood Kit (Ambion, Life Technologies, Paisley, UK) according to the manufacturer's instructions. cDNA samples were obtained with Superscript^TM^ First-Strand Synthesis System for RT-PCR (Invitrogen, Life Technologies, Carlsbad, California, USA).

Quantitative expression experiments were performed in the control group and in family members following MIQE recommendations (14). The efficiency analysis of primers was previously performed for *TPS* (tryptase gene) forward 5′-GCGATGTGGACAATGATGAG-3′ and reverse 5′-TTCCATTATGGGGACCTTCA-3′ primers as well as for *TBP* (TATA box binding protein) forward 5′-ATAGGGATTCCGGGAGTCAT-3′ and reverse 5′-GAACATCATGGATCAGAACAACA-3′ primers (*TBP* was used as reference). Relative quantitative PCR (qPCR) was performed in a LightCycler 480 Instrument and using SYBR Green I Master (Roche, Indianapolis, IN, USA). The luminescence signal acquisition was obtained in the last cycle of amplification (unique acquisition) and in the melting curve (continuous acquisition). Two negative control samples were included and three replications per sample were performed.

Expression differences were calculated by the Livak method (15). The process was carried out in an accredited laboratory following ISO-15189.

### Gene-Dose Analysis

A relative qPCR was carried out to determine the gene-dose of *TPSAB1, TPSB2*, and *TPSD1* genes. The efficiency of *TPSAB1* primers: forward 5′-CCAAAACACCACTGCTTCCT-3′ and reverse 5′-AGGATAGGGAAGGGTCCTCA-3′; *TPSB2* primers: forward 5′-CAGCGAGTGGGCATCGTT-3′, and reverse 5′-TGCATCCAGTATCGGTCGC-3′, and *TPSD1* primers: forward 5′-GGGGTTTGGAGAGTCCCTTA-3′ and reverse 5′-TCCACATAGCAAGTCCGTGA-3′ was tested by generation of a standard curve with serial dilutions, using the slope of the regression line [*E* = 10(1–slope)]. Reactions were performed in triplicate in a final volume of 15 μl, including 50 ng of genomic DNA, SYBR Green I Master (Roche Applied Science, IN, USA), and 0.4 μM of each primer. Amplification was performed using the LightCycler 480 Instrument (Roche Applied Science, IN, USA). The qPCR protocol used an initial denaturation step (95°C for 10 s) followed by amplification and quantification steps repeated for 45 cycles (95°C for 30 s, 60°C for 30 s, and 72°C for 30 s). *GAPDH* was considered as the reference gene and the copy number was estimated from the normalized ratio 2 × 2–(ΔΔCt) using a DNA control obtained from a healthy subject. qPCR product quality was monitored using post-PCR melt curve analysis.

### Tryptase Gene Genotyping

Genotyping of the *TPSAB1* gene was performed by quantitative real-time digital PCR (qdPCR) using the Biomark™ HD system (Fluidigm, South SanFrancisco, CA, USA). To detect copy number variation (CNV) in α and β alleles, specific probes, as previously described in Lyons et al. (8), were used for qdPCR reaction according to the 37 K digital PCR protocol (Fluidigm). Data were analyzed using Digital PCR Analysis software (Fluidigm). Hereditary α-tryptasemia (HαT) was defined as three or more α-tryptase copies or two α-tryptase copies in the presence of three β-tryptase copies.

### Data Analysis

The databases consulted to analyze the *Tryptase* genes were the Genome Browser of California University (http://genome.ucsc.edu/), Aceview (http://www.ncbi.nlm.nih.gov/IEB/Research/Acembly/), Genecards (http://www.genecards.or), and NCBI from the National Center for Biotechnology Information (http://www.ncbi.nlm.nih.gov/projects/SNP/).

Statistical analysis was performed using SPSS 19.0 software (Chicago, Illinois, USA), the statistical power was analyzed with available software in http://www.dssresearch.conm/toolkit/sscalc/size_a2.asp and http://statpages.org/proppowr.html.

## Results

We had the opportunity to study four members of a family with elevated BST for 16 years. The four members of the same family were the index case (I.2), her sister (I.3), and her two daughters (II.1 and II.2). BST values were normal in her husband (I.1) and controls (Figure 1 and Table 1). Additionally, *KIT* mutations were negative, and a clonal MCD was discarded after the performance of a BM biopsy. Furthermore, the patient has been followed for 14 years, and has been asymptomatic for the last 9 years (except for pruritus sine materia) despite the persistence of elevated BST (Table 1). The rest of the family has also been asymptomatic all this time. We consider that this family with elevated BST, in the absence of clinical symptoms, and without clonal MCs, has an asymptomatic HαT.

**Table 1:** Family clinical features.

	**BST (mcg/ml)**	**Symptoms**	**Increased BST during crisis (mcg/ml)**	**Response of clinical symptoms to HRB**
I.2	59.9 (2003), 62.2 (2004), 46.4 (2005), 50.2 (2008)	Episodes of abdominalgia, vomiting, chills, hypothermia, syncope, metabolic acidosis, and hypokalemia	No	No
II.1	30.5	Asymptomatic	–	
II.2	28.5	Asymptomatic	–	
I.3	46.0	Asymptomatic	–	
I.1	4.22	Asymptomatic	–	

*BST, Basal serum tryptase levels*.

We aimed to interrogate whether BST could correlate with mRNA expression and gene dose in this family. We detected an increase in the mRNA expression of tryptase genes in the family members compared to controls. Thus, patient I.2 showed a dramatic increment in fold change compared to control average (Table 2). Both patients I.2 and I.3 showed BST > 20 ng/ml, which was higher (average 54.67 ng/ml) for I.2 than for I.3 (46 ng/ml). The index case also presented the highest levels of tryptase gene expression. These levels correlated with a highly increased tryptase mRNA expression in those patients (Pearson 0.977, *p* = 0.004).

**Table 2:** *TPSAB1* mRNA expression levels.

**Patient**	**ΔΔCt**	**Fold**
I.2	−5.09	33.99
I.3	−4.17	17.96
I.1	1.76	0.29
Control #1	−0.99	1.99
Control #2	−0.44	1.35

In addition, we detected an increase of the *TPSAB1* gene dose in the family members, which correlated with BST levels (Pearson 0.868, *p* = 0.011), whereas *TPSB2* and *TPSD1* showed a normal gene dose. The index case (I.2) and their relatives I.3, II.1, and II.2 had a higher copy number than the controls for *TPSAB1*. Considering two as the normal number of copies, we found: patient I.2 = 4.13; patient I.3 = 4.47; patient II.1 = 4.57; and patient II.2 = 4.04. These results of CNV were confirmed, with qdPCR revealing a hereditary copy number gain genotype (3β2α) present in all family members studied (Table 3).

**Table 3:** Identified tryptase genotypes encoded at *TPSAB1* and *TPSB2*.

**Patient**	**Genotype**	**Phenotype**
I.2	3β2α	Hereditary α-tryptasemia
I.3	3β2α	Hereditary α-tryptasemia
II.1	3β2α	Hereditary α-tryptasemia
II.2	3β2α	Hereditary α-tryptasemia
I.1	3β1α	Conserved *TPSAB1* copy number
Control	3β1α	Conserved *TPSAB1* copy number

## Discussion

We found four members of the same family with elevated BST that were followed-up for 16 years. The index case presented the highest BST and some symptoms that could be compatible with atypical symptoms of the release of MC mediators, but the patient did not fulfill all the diagnostic criteria of MCAS (3). Additionally, a clonal mast cell disorder was discarded in the index case by performing a BM biopsy, and *KIT* mutations studied were negative. Some cases of familial mastocytosis have been described (16–19). Molderings et al. (20) described a familial occurrence of systemic MC activation disease reporting that mutated disease-related operator and/or regulator genes could be responsible for the development of somatic mutations in *KIT* and other proteins involved in the regulation of mast cell activity. Recently, the entity known as hereditary alpha-hypertryptasemia has emerged. Lyons et al. (21) identified nine atopic subjects with persistent increases in BST in the absence of evidence for a clonal MCD. They found an autosomal dominant inheritance pattern of increased BST, which is in agreement with our family. Although they observed an increase in MC numbers on BM, none of the five index patients that underwent BM biopsy met the World Health Organization established criteria for the diagnosis of systemic mastocytosis (22) or the ones proposed for monoclonal MCA (3). Nevertheless, all of the patients were symptomatic. In addition, these authors also found that elevated BST identified a multisystem disorder associated with increased *TPSAB1* copy number (8). Finally, Sabato et al. (23) reported a family with elevated BST and MCAS. They found elevated BST in seven relatives of three consecutive generations, suggesting a monogenic form of hypertryptasemia with autosomal dominant inheritance. Two patients were completely asymptomatic and a third one only had apathy and migraine with a normal BM biopsy. These three patients are similar to the two daughters (II.1, II.2) and the sister (I.3) of our index case.

We consider that this family with high BST in the absence of clinical symptoms and without clonal MCs has an asymptomatic familial hypertryptasemia or HαT. We detected an increase in the mRNA expression of tryptase genes in the family members compared to controls. The index case also presented the highest BST levels which correlated with a highly increased tryptase mRNA expression. In addition, we detected an increase of *TPSAB1* gene dose in patients which correlated with high BST levels in those patients. This agrees with Sabato et al. (24) that recently reported a *TPSAB1* quintuplication in a highly symptomatic patient with clonal MC disease, remarking that individuals carrying three or more copies of α-tryptase had higher BST and more symptoms than those carrying two copies.

We performed CNV analysis revealing a hereditary copy number gain phenotype (3β2α) present in the index case (I.2), her sister (I.3), and her daughters (II.1 and II.2). Although the mRNA of patients II.1 and II.2 was not available, a good correlation between gene expression and BST levels was shown in the rest of the samples. Finally, other factors capable of modulating gene expression can be involved, such as epigenetic factors (25).

In conclusion, we present a family with HαT, elevated BST, and mRNA gene expression in the absence of MC clonality. Unlike what has been reported to date, nearly all of them have remained asymptomatic during a long follow-up period. Typically, BSTs have been associated with having an MCD. However, HαT associates with increased copy numbers of the *TPSAB1* gene, which leads to high BSTs (4) and may not be associated with the presence of usual MC-mediator release symptoms or may even be asymptomatic. Since an estimated 4–6% of the population has high levels of BST (26, 27), HαT should be taken into account in the differential diagnosis of patients with symptoms of mastocyte activation and increased BST levels, not only to achieve a correct diagnosis and treatment but also to advise on prognosis and recommendations.

## Data Availability Statement

The raw data supporting the conclusions of this article will be made available by the authors, without undue reservation.

## Ethics Statement

The studies involving human participants were reviewed and approved by the local Ethics Committee of the University Hospital of Salamanca (PI120913) and an informed consent was signed in all cases. The patients/participants provided their written informed consent to participate in this study.

## Author Contributions

LH-H, CS, ID, and MI-G conceived and planned the experiments. LH-H, EM-V, and AG-S carried out the experiments. EM, FL, and ID collect the samples. LH-H, CS, EM-V, AG-S, DG-O, ID, and MI-G contributed to the interpretation of the results. All authors provided critical feedback and helped shape the research, analysis, and manuscript. All authors agree to be accountable for the content of the work.

## Conflict of Interest

The authors declare that the research was conducted in the absence of any commercial or financial relationships that could be construed as a potential conflict of interest.

## Abbreviations

BST: basal serum tryptase
HAT or HαT: hereditary α-tryptasemia
BM: bone marrow
MC: mast cell
MCD: mast cell disorder
MCAS: mast cell activation syndrome.
